# Suggestive Serological Evidence of Infection with Shrew-Borne Imjin Virus (*Hantaviridae*) in Humans

**DOI:** 10.3390/v11121128

**Published:** 2019-12-06

**Authors:** Rui Qi, Xi-Feng Sun, Xiang-Rong Qin, Li-Jun Wang, Min Zhao, Fachun Jiang, Ling Wang, Xiao-Ying Lei, Jian-Wei Liu, Xue-Jie Yu

**Affiliations:** 1 State Key Laboratory of Virology, School of Health Sciences, Wuhan University, Wuhan 430071, Chinaliujianwei@whu.edu.cn (J.-W.L.); 2 School of Public Health, Shandong University, Jinan 250012, China; 3 Qingdao Center for Disease Control and Prevention, Qingdao 266033, China; 4 Zibo Center for Disease Control and Prevention, Zibo 255026, China

**Keywords:** hantavirus, Imjin virus, shrew

## Abstract

The pathogenicity of the shrew-borne Imjin virus (MJNV) is unknown. The objective of our study was to find serological evidence of MJNV infection in humans. Partial MJNV nucleocapsid protein (NP) was cloned and expressed as an antigen for double-antigen sandwich ELISA, IgM capture ELISA, and dot blot to detect MJNV specific antibodies in hemorrhagic fever with renal syndrome (HFRS) patients’ and healthy persons’ sera from endemic areas in China. The purified recombinant NP reacted with neither the 90 healthy individuals’ sera from non-endemic areas of MJNV nor the 100 antisera to HFRS-causing virus, indicating that the MJNV NP had no cross-reaction with normal human sera and HFRS-causing viral antibodies. As determined by screening ELISA and dot blot analysis, IgG antibodies against MJNV NP were detected in sera from two of 385 healthy individuals from MJNV-endemic areas, suggesting infection with MJNV or MJNV-like thottimvirus. Based on the suggestive evidence, healthcare workers should be alert to febrile diseases occurring among individuals with exposure to shrew-infested habitats.

## 1. Introduction

Members of the family *Hantaviridae* are enveloped negative-stranded RNA viruses with a tripartite genome consisting of large (L), medium (M), and small (S) RNA segments, respectively [1,2]. The latest taxonomical proposal lists approximately 50 hantavirus species [3], which are hosted by mammalian species of the orders Rodentia, Eulipotyphla, and Chiroptera [4,5], with occasional spillover into humans. Hantavirus cardiopulmonary syndrome (HCPS) is the primary form of hantavirus syndrome in the New World [6]. Hemorrhagic fever with renal syndrome (HFRS) occurs in the Old World with most HFRS cases (approximately 90%) having been reported in China [7]. However, increasing evidence points to the existence of HFRS in the New World due to the local presence of infected rats [8]. In the past decades, more than 10,000 HFRS cases were reported annually, and the fatality rate was about 1% in China [9].

Previously, in the rural population of two prefecture-level cities (Zibo and Qingdao) of China, we found HFRS had a high incidence rate which ranged from 1.96 cases/100,000 persons to 28.9/100,000 in different years [10,11]. These studies indicated HFRS was epidemic in these areas. Peridomestic rodents were also captured for orthohantavirus detection in the two cities and orthohantavirus antigens were found by immunofluorescence assay in 5.2% (29/559) and 3.8% (9/240) of these rodents, respectively [10,11], and the seropositive rate to hantavirus of host animals was as high as 23.3% [12]. Hantaan orthohantavirus (HTNV) and Seoul orthohantavirus (SEOV) are the causative agents of HFRS in these areas. A notable fact was that the captured small animals included not only rodents but also shrews. A total of 178 (15.8%) *Crocidura* shrews were accidentally captured in the same areas as rodents, simultaneously [13]. Reverse transcription-polymerase chain reaction (RT-PCR) was used for amplifying hantaviral RNA in these shrews. The results showed that 2 of 178 (1.1%) shrews (2 of 164 *Crocidura lasiura*, 0 of 7 *Crocidura attenuata*, and 7 *Crocidura shantugensis*) were PCR positive to SEOV. In addition, 10.7% (19/178) of these shrews were positive to a newly discovered shrew-borne Imjin virus (MJNV) in Korea and China [13,14]. However, it is not clear whether MJNV can infect humans. The objective of our study was to find serological evidence of MJNV infection in humans through an investigation of the presence of anti-MJNV antibody in healthy humans and HFRS patients.

## 2. Materials and Methods

### 2.1. Sample Collection

Sera of healthy persons living in epidemic areas were collected for MJNV antibody detection. These healthy persons were from a population at high risk of hantavirus infection in rural areas of Qingdao City, Shandong Province, China. These persons volunteered for vaccination and samples were collected before vaccination. In addition, a total of 90 sera from healthy persons from non-endemic areas of MJNV and 227 acute sera of clinically diagnosed HFRS patients were collected. Healthy persons from non-endemic areas were university students that lived in cities and not in rural areas. Sera from clinically diagnosed HFRS patients were collected from hospitals under the jurisdiction of Zibo and Qingdao cities. Clinical diagnosis of these patients was based on the HFRS national surveillance program criteria developed by the Chinese Centre for Diseases Control and Prevention (CDC). (http://www.chinacdc.cn/jkzt/crb/lxxcxr/cxrjc/200508/t20050810_24189.htm). These HFRS sera had not been confirmed by laboratory tests when collected. Commercial ELISA Kits (Wantai Biological Pharmacy, Beijing, China) were used to confirm the HFRS-causing orthohantavirus infection in this study. This commercial kit can detect antibodies against HTNV and SEOV but cannot differentiate between them (cross-reaction). The kit was only used to exclude anti-HTNV or -SEOV sera from study samples.

The study was reviewed and approved by the ethics committees of Wuhan University (2018010). Written informed consent was obtained from each person.

### 2.2. Expression of Recombinant MJNV NP

We did a BLAST search analysis to identify the unique sequence of MJNV NP. The N-terminal of MJNV NP (amino acids 1 to 124) was selected as an antigen to detect antibodies against MJNV in humans based on the fact that it is conserved among strains of MJNV, but has very low similarity (35%) to the corresponding sequence in HTNV and SEOV. The DNA sequence of MJNV NP N terminal corresponding to amino acids 1 to 124 and the full-length MJNV NP gene were amplified from an MJNV positive shrew collected previously from Qingdao City (GenBank: ARA95707.1) (*11*). The N-terminal NP was tagged with a six-histidine and cloned into the pET-22b (+) vector while the full-length NP was tagged with GST and cloned into the pGEX-KG vector. All recombinant proteins were expressed in *E. coli*. The N-terminal NP was purified through a nickel column, size exclusion chromatography column, and anion exchange chromatography (Amersham GE Health, Uppsala, Sweden) with the AKTA system (GE Health, Uppsala, Sweden). The full-length NP was only used for mouse immunization and was purified only through the GST affinity column and separated from gel electrophoresis before immunization. The purity and identity of the protein were analyzed by SDS-PAGE and Western blotting.

### 2.3. Immunization of Mice

The full-length recombinant MJNV NP expressed in *E. coli* was separated by electrophoresis on 12% gel. After electrophoresis, the gel was soaked in 0.1 M KCl for about 5 min until protein bands appeared as white precipitates against a clear gel background. Then the bands were cut out from the gel and suspended by homogenizing the gel slices in a minimum volume of phosphate-buffered saline (PBS) using a tissue grinder. The protein-gel suspension was used to immunize Kunming mice (purchased from Hubei Center for Disease Control and Prevention, Wuhan, China). Each mouse was intraperitoneally injected with 400 µl of gel suspension and boosted once after 3 weeks. A month later after the first injection, mice were bled, and sera were obtained. The immune mouse sera were used as positive controls for ELISA.

### 2.4. ELISA

For the double-antigen sandwich ELISA, the purified protein not only coated plates as the binding antigen but was also used as the detecting antigen to visualize the results when conjugated with horseradish peroxidase (HRP). HRP was conjugated by HRP Conjugation Kit (GalaxyBio, Beijing, China). The optimal working concentrations of coating antigen, serum, and HRP-conjugated antigens were determined by the checkerboard titration method. Purified partial MJNV NP was diluted with three concentrations (500 ng/100 μL, 100 ng/100 μL, and 10 ng/100 μL) of 0.05 M Na bicarbonate/carbonate buffer (pH 9.6) in coated high-binding 96-well microtiter plates (Costar, Corning, NY, USA) at a volume of 100 μL per well at 4 °C for 24 h. The positive and negative sera were serially diluted 10-fold from 1:10 to 1:1000 in PBS. HRP-conjugated protein was serially diluted 10-fold from 1:1000 to 1:8000 in PBS Tween-20 (PBST).

For IgM capture, ELISA plates coated with monoclonal anti-human IgM antibody (μ-chain specific) were kindly donated by Dr. Jiao (Jiangsu CDC, China). Checkerboard titrations for sera and HRP-conjugated antigen were the same as the double-antigen sandwich ELISA. 

Each condition of checkerboard titration was made in duplicate. Plates were blocked with 250 μL of 5% dried skim milk in 0.01 mM PBST (pH 7.4) at 37 °C for 2 h. The incubation of sera and HRP-conjugated protein were 60 min and 30 min, respectively, at 37 °C. Washes between procedures were 5 rounds with PBST. To visualize the results, 50 μL/well of 3,3,5,5-tetramethylethylenediamine solution (TMB) (PR1200, Solarbio, Beijing, China) was added and the plates were incubated for less than 10 min at 37 °C. The reaction was stopped by the addition of 50 μL 2 M sulfuric acid, and the optical density (OD) value of each ELISA was read using an ELISA reader at 450 nm. The conditions that were considered optimal have a positive serum OD_450_ value close to 1.0 and negative serum OD_450_ less than 0.1. The mean and standard deviation (SD) for OD_450_ of negative sera were deemed to be the cutoff value for OD_450_ of positive sera.

### 2.5. RT-PCR 

RT-PCR was used to amplify MJNV RNA in the hantavirus-negative patients and MJNV-positive healthy persons as described previously [13].

### 2.6. Dot Blot and Western blot

Purified MJNV NP N-terminal protein (1 μL) was spotted onto a nitrocellulose (NC) membrane. After being air-dried, the membrane was blocked with 5% dried skimmed milk in Tris-Buffered Saline Tween-20 (TBST) and then incubated with sera followed by detecting for antigen conjugated to HRP. Five rounds of washes with TBST occurred between incubations. After being incubated and washed, the chemiluminescent substrate was added and the membranes were imaged using equipment (Amersham Imager 600, GE, Uppsala, Sweden). For the Western blot, the expressed recombinant protein was separated using SDS-PAGE electrophoresis (5% stacking gel and 12% separating gel), followed by electroblotting (200 mA, 1 h) which made the protein move from within the gel onto an NC membrane. Procedures for membrane blocking, washing, incubation, and visualization were the same as the dot blot. When Western blot was used for demonstrating the expression of MJNV NP N-terminal protein, the anti-6HIS antibody conjugated with HRP was used as the primary and detection antibody. Purified MJNV NP protein conjugated with HRP was used as the secondary antibody for antisera detection.

## 3. Results

### 3.1. Establishing ELISA Diagnostic Assay with MJNV NP

**Figure 1:**
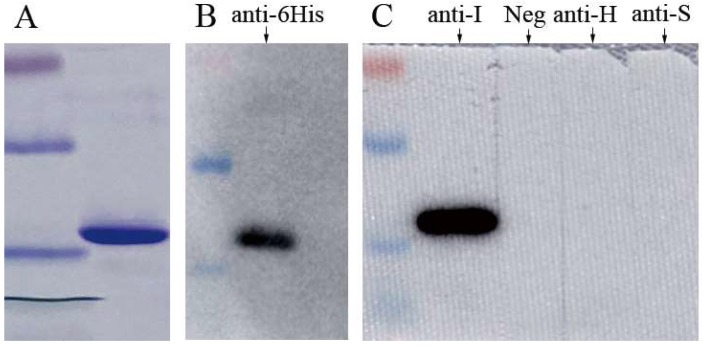
Legend: (**A**) The purified recombinant MJNV NP N-terminal on SDS-PAGE and stained by Coomassie brilliant blue. (**B**) Western blot of the recombinant MJNV NP N-terminal reacted with an anti-his tag antibody. (**C**) Western blot of the recombinant MJNV NP N-terminal reacted with mouse antisera immunized with recombinant full-length MJNV NP (anti-I), negative-control (Neg), HTNV (anti-H), and SEOV (anti-S) positive samples. Negative control was healthy human serum; HTNV or SEOV were positive sera stored in our laboratory confirmed by PCR, previously.

The partial MJNV NP was successfully expressed and purified. Only one band was clearly visible in the result of Coomassie brilliant blue-stained SDS-PAGE (Figure 1A). The approximate molecular masses of the band were the same as theoretical molecular masses (about 16 kDa), and this band could be recognized by antibodies against histidine-tag at the end of this target protein (Figure 1B). The partial MJNV NP was successfully detected with positive control of mouse sera against the full-length NP (Figure 1C). The result indicated the recombinant MJNV NP was effective in the detection of antibodies against MJNV. In the subsequent assays, this NP was used to detect the antibody against MJNV in the serum. The optimal working condition for the double-antigen sandwich ELISA was 100 ng/100 μL (well) of the coating antigen combined with 1:100 dilution of serum sample and 1:8000 diluted HRP-conjugated antigen, which gave an OD_450_ value of around 1.0 for the positive-control serum and 0.06 for the negative-control serum. The optimal working conditions of HRP-conjugated antigen and serum were the same for IgM capture ELISA.

### 3.2. Specificity of MJNV NP

We tested the specificity of MJNV NP N-terminal by reaction with sera of healthy persons from non-epidemic areas. ELISA showed that none of the sera from the 90 healthy persons reacted with the recombinant MJNV NP N-terminal. 

The commercial ELISA Kit for HFRS showed that 100 of 227 (44.1%) clinically diagnosed HFRS patients were positive to HFRS with 56 patients IgM positive, 27 IgG positive, and 17 positive to both, suggesting these patients were infected with HTNV or SEOV orthohantavirus. The sera of 127 HFRS patients were negative to HTNV or SEOV, suggesting these patients were misdiagnosed as HFRS. Double-antigen sandwich ELISA and RT-PCR were used to test MJNV antibodies and RNA in all 227 HFRS patients’ sera and none of the sera were positive to MJNV, suggesting that none of these patients were infected with MJNV regardless if they were positive or not to HFRS-causing hantaviruses (HTNV or SEOV). The above results suggested that the recombinant MJNV NP did not cross-react with normal human immunoglobulins and HTNV or SEOV stimulated antibodies. It also suggested that none of the misdiagnosed HFRS cases was caused by MJNV. 

### 3.3. Prevalence of MJNV in Endemic Area

**Figure 2:**
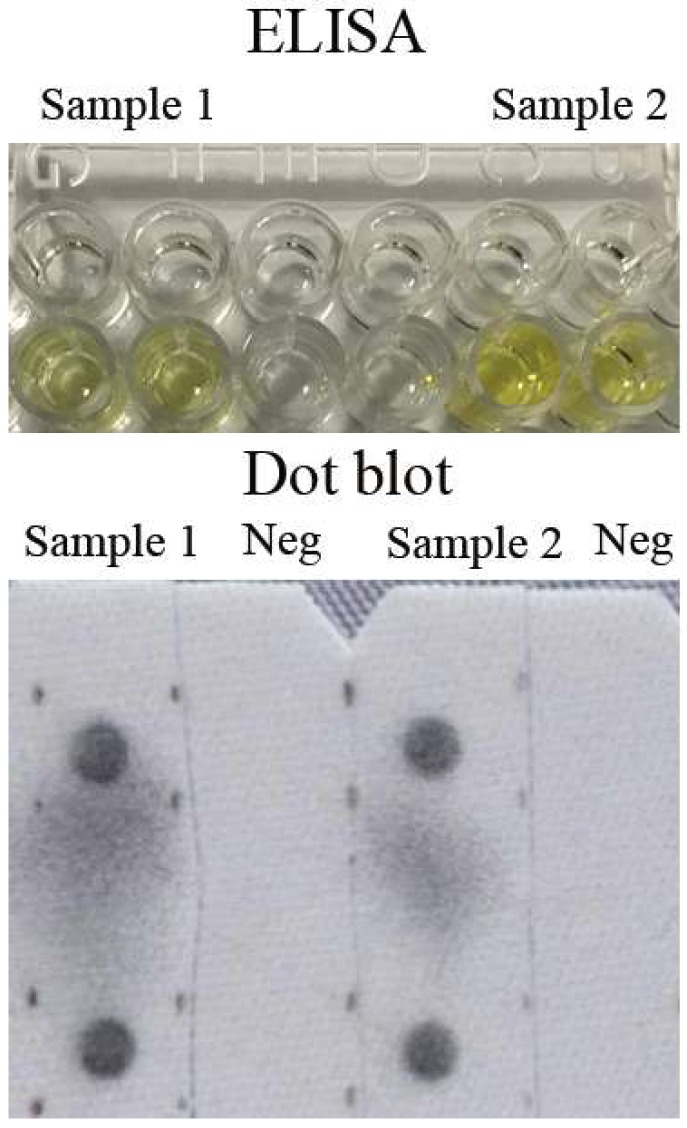
Legend: Repeated results of two positive human sera detected by double-antigen sandwich ELISA and dot blot. Each sample was detected in duplicate each time and, both, ELISA and dot blot were repeated twice. Neg: negative–control sample from a healthy human.

A total of 385 healthy persons’ sera were obtained from farmers living in an MJNV endemic area in Qingdao City for detecting antibodies against MJNV. Among these people, 72.2% (278/385) were men and 27.8% (107/385) were women. Approximately 70% (271/385) were between 30 and 50 years of age (ranged from 26 to 60). Double-antigen sandwich ELISA showed that 2 (0.5%) of these sera were positive to the MJNV antibody (IgG) (Figure 2). The reactions of the two sera with MJNV NP were further repeated by dot blot (Figure 2). Both ELISA and dot blot experiments were repeated twice with duplicate each time. All experiments returned consistent positive results, indicating the results were reliable. The 2 sera were also negative to IgM and IgG antibodies for HFRS-causing hantaviruses, which was confirmed by commercial ELISA kits. IgM capture ELISA and RT-PCR were then used to detect IgM antibodies against MJNV and its RNA. Both results were negative, meaning those two persons were not acutely infected with MJNV. These results are suggestive serological evidence of MJNV infection in humans in MJNV-endemic areas. Both affected farmers were male and aged 38 and 48 years old, respectively.

## 4. Discussion

No commercial kit was available for MJNV immunoassay, therefore, all antigens and immunoassays for MJNV were developed in this study. The aim of this study was to find serological evidence of MJNV infection in humans, so the specificity was of particular consideration to this study to avoid false positives. Double antigen sandwich immunoassay was used because they are highly specific as the antigen needs to bind the specific antibody twice. However, double-antigen sandwich immunoassay can only indistinguishably detect total antibodies (IgM and IgG), so IgM capture ELISA coated with monoclonal anti-human IgM antibody (μ-chain specific) was then used to distinguish IgM antibody. In addition to ELISA, dot blot was also used to confirm the results of positive MJNV sera.

Both ELISA and dot blot showed that the MJNV antibody was detected in healthy persons from MJNV-epidemic areas in China. The results are suggestive of serological evidence of MJNV infection in humans. The study areas of Qingdao City are distributed over 3449 km^2^ of land with a total population of 1.53 million, of whom 1.43 million (93.5%) lived in rural areas (http://qdsq.qingdao.gov.cn/n15752132/n20546841/n20641803/n32565526/181113155057473377.html). A previous study showed that the density of hantavirus hosts (rodents and shrews) in the whole rural area of Qingdao City was 3.7%. Of those hosts, approximately 50% were trapped in residential homes and the proportion of shrews was 8.4% [15]. It showed that shrews were peridomestic, frequently living within or in close proximity to human dwellings in Qingdao. Our previous study reported that in the areas where serum samples were collected for this study, the rate that shrews carried MJNV was about 10% [13]. MJNV antibodies were detected in healthy people from MJNV endemic areas by ELISA and dot blot and if the results can be confirmed by the gold-standard plaque-reduction neutralization (PRNT) test in future studies, it would demonstrate that MJNV could infect people. Even if our results are confirmed by PRNT in the future, it is still not clear whether MJNV will cause severe disease. We assumed that misdiagnosed HFRS patients who were HTNV and SEOV negative might be infected with MJNV. However, the 127 misdiagnosed HFRS cases were all negative to MJNV antibody and RNA, suggesting either the sample size was too small or MJNV was causing disease with different clinical manifestations than HFRS. 

The resulting evidence from our patients’ sera was insufficient to suggest that MJNV did not cause any disease. Firstly, patients included in this study were based on the hypotheses that MJNV might cause HFRS like HNTV and SEOV. If MJNV caused another disease instead of HFRS, it would be normal that no antibodies against MJNV would be detected in these clinically diagnosed HFRS patients. As mentioned in a previous study [14], no one had the prescience to predict that hantaviruses could cause diseases other than HFRS before a hantavirus, Sin Nombre virus was found that could cause HCPS. If hantaviruses could be capable of causing diseases as clinically disparate as HFRS and HCPS, we could not exclude the possibility that the new shrew-borne hantavirus, MJNV, might be pathogenic to humans but cause a disease that was clinically distinct from HFRS. There might be another probability that MJNV caused a mild disease of low concern, such as a spontaneously transient flu-like illness with the absence of severe symptoms, such as kidney function impediment, which has also been described recently for SEOV-induced HFRS [8]. Whether MJNV causes severe disease in humans needs to be further investigated. 

Nucleocapsid protein of MJNV consisted of about 430 amino acids. Compared with HTNV and SEOV, the amino acid sequence of the NP of MJNV was different by more than 50% (identities were 46.8% and 45.6%, respectively) [14]. The N-terminal 124 amino acids of MJNV NP were used to detect serum antibodies in our study because it has been reported that the N-terminal part of NP of hantaviruses bore immunodominant epitopes. Some studies using monoclonal antibodies (MAbs) against NP of hantaviruses (such as Hantaan virus, Puumala virus, Andes virus, Carizale virus, and Thottapalayam virus) showed that the binding region of MAbs was the N-terminal parts of NP [16,17,18,19,20]. Similarly, investigations of antibody epitopes induced in HFRS and HCPS patient sera and rodent-borne orthohantaviruses-infected animals using a synthetic peptide antigen and/or partial antigens were reported [16,21,22,23,24,25]. These studies showed that immunodominant epitopes of NP were also present in the N-terminal region even in these polyclonal antibodies. These results indicated that antibodies against NP were mostly produced against the N-terminal region of NP of hantaviruses. The immunodominant N-terminal region of NP of hantavirus could be useful for detecting antibodies in serological diagnoses. We demonstrated that the NP of MJNV did not cross-react with HTNV and SEOV. If there is no other MJNV-like or nucleocapsid protein antigenically related thottimvirus, it could be a good candidate for serodiagnosis of MJNV infection in animals and humans. The absence of a neutralization test was a limitation of our study as we do not have MJNV. Previously, Yanagihara and colleagues tested 2,732 sera from patients with acute febrile illnesses and showed that 3 patients were determined as anti-MJNV positive by ELISA, IFA, and Western blot. However, all 3 sera failed to be positive by PRNT [26]. The results determined by immunoassays like ELISA can only be used as suggestive serological evidence. Evidence from gold-standard tests like PRNT is required in a future study to serve as definitive proof of MJNV infection in humans. 

## 5. Conclusions

In conclusion, as determined by screening ELISA and dot blot analysis, IgG antibodies against MJNV NP were detected in sera from two of 385 healthy individuals from MJNV-endemic areas, suggesting infection with MJNV or MJNV-like thottimvirus. Based on the suggestive evidence, healthcare workers should be alert to febrile diseases occurring among individuals with exposure to shrew-infested habitats.
